# Remote multicomponent rehabilitation compared to standard care for survivors of critical illness after hospital discharge (iRehab): a protocol for a randomised controlled assessor-blind clinical and cost-effectiveness trial

**DOI:** 10.3310/nihropenres.13910.2

**Published:** 2025-08-22

**Authors:** Brenda O'Neill, Judy Martina Bradley, Bronwen Connolly, Julie Bruce, Martin Underwood, Ranjit Lall, Chen Ji, Jill Costley, Rachel Clarke, Paul Dark, Penelope Firshman, Nigel D Hart, Annette Henderson, Katherine Jones, Roger Kenyon, Jason Madan, Gavin D Perkins, Mariam Ratna, Kerry Raynes, Ella Terblanche, Rowena Williams, Mandana Zanganeh, Danny McAuley

**Affiliations:** 1 Ulster University Institute of Nursing and Health Research, Belfast, Northern Ireland, UK; 2 Queen's University Belfast Wellcome-Wolfson Institute for Experimental Medicine, Belfast, Northern Ireland, UK; 3 The University of Melbourne Melbourne School of Health Sciences, Melbourne, Victoria, Australia; 4 University of Warwick Warwick Clinical Trials Unit, Coventry, England, UK; 5 University Hospitals Coventry and Warwickshire NHS Trust, Coventry, England, UK; 6 University Hospitals Plymouth NHS Trust, Plymouth, England, UK; 7 The University of Manchester Division of Infection Immunity and Respiratory Medicine, Manchester, England, UK; 8 Surrey and Sussex Healthcare NHS Trust, Redhill, England, UK; 9 General Practitioner and Clinical Professor in General Practice, School of Medicine, Dentistry and Biomedical Sciences, Queen's University Belfast, Belfast, Northern Ireland, UK; 10 Patient advisory group and PPI representative on iRehab Trial Management Group, Preston, England, UK; 11 Principal Critical Care Dietitian, Health Sciences University, Bournemouth, England, UK

**Keywords:** Intensive Care, ICU, Rehabilitation, Recovery, Critical illness, Individualised, Remote

## Abstract

**Background:**

The consequences of critical illness can be substantial and multifactorial, encompassing physical deconditioning, mental health impairments, fatigue, and declines in health-related quality of life. We hypothesise that for people discharged after intensive care unit (ICU) for a critical illness, a six-week remote multicomponent rehabilitation intervention improves health-related quality of life, physical function, fatigue, mood, and other health-related outcomes after eight weeks, compared to standard care.

**Methods:**

This is a pragmatic, randomised controlled, open-label, assessor blind, multicentre, clinical and cost effectiveness trial with internal pilot and embedded process evaluation. Recruitment will take place in NHS hospitals across the UK. Adults (n=428: control n= 197; intervention: n=231) within 12 weeks of discharge from hospital following an ICU admission for critical illness, requiring mechanical ventilation ≥48hours will be recruited.

The intervention is a six week multicomponent, structured, rehabilitation programme, delivered remotely by a trained intervention team. The intervention includes four components: weekly symptom management; targeted exercise; psychological support, and peer support and information. The control group will receive standard NHS care.

The primary outcome is Health-related quality of life (HRQoL) at eight weeks post-randomisation measured using the EQ-5D-5L. Secondary outcomes are: HRQoL (six months), physical function, fatigue, illness perceptions anxiety and depression, healthcare resource use at eight weeks and six months and intervention acceptability.

**Conclusions:**

This trial will test a centrally delivered mulitcomponent rehabilitation intervention for survivors of critical illness, irrespective of geographic location or critical illness diagnosis.

**Trial registration:**

The trial is registered (04.07.2022) with the International Standard Randomised Controlled Trial Number (ISRCTN) Register ISRCTN11266403 https://doi.org/10.1186/ISRCTN11266403

## Plain Language Summary

**Background:**

Patients treated in intensive care need a great deal of special care and support. After discharge from hospital, some people find their muscles are still weak and their ability to exercise and to do everyday things may still be affected. They can also have confused memories of their time in the intensive care unit. Currently, in most areas in the UK there is no organised rehabilitation offered to patients after discharge home following critical illness

**Purpose:**

We want to find out if a six-week rehabilitation programme (support and exercises) will help patients following intensive care who are discharged from hospital.

**Methods:**

We will include patients (n=428) who have gone home from hospital after critical illness. In our previous research studies, patients helped us to identify what should be included in a support and exercise rehabilitation programme. The programme is based on what the patients’ needs are. A member of the healthcare team will speak to every individual patient by video or phone on a weekly basis. They will provide helpful information, tips to support recovery, and help to do some exercises.

**Measurements:**

In this study we will compare the quality of life, physical strength, and emotional wellbeing of the patients who take part in the rehabilitation programme with the patients who do not. We will also ask patients about their tiredness, and views about recovery. This information will be collected at 6 weeks and at 6 months by researchers not involved in the rehabilitation. Additionally, we will be looking at value for money.

**Proposed benefits:**

We hope that patients will be helped by this rehabilitation programme. If successful, then it will provide useful information to help the development of cost-effective services for patients after critical illness. At the end of the trial, we will share our findings.

## Background

Each year, in England, Wales, and Northern Ireland, around 130,000 survivors of critical illness are discharged from hospital ^1^. The consequences of critical illness are substantial and multifactorial, often referred to as Post Intensive Care Syndrome (PICS). PICS encompasses physical deconditioning, respiratory and swallowing problems, reduced activities of daily living, cognitive and mental health impairments, fatigue, and poor health-related quality of life (HRQoL) ^2– 8^. In the UK, one in four people recovering from critical illness experience an unplanned hospital readmission within 90 days of discharge ^9^. Nearly half of survivors of a critical illness fail to return to work after 12 months ^10, 11^. UK data also highlight the increase in social care support required for ICU survivors ^11^. Therefore, there is a need to intervene to improve the long-term health of patients discharged home after intensive care ^12^.

Although regular assessment and rehabilitation is recommended as people transition between care settings and different stages of recovery, in the UK these services are ad hoc, and variable in terms of structure, content, and format of delivery ^13^. A national survey of UK ICUs found that very few hospitals offer a post-discharge physical rehabilitation programme or structured support (31/176, 18%) ^14^.

Exercise programmes can aid physical recovery and support emotional and mental wellbeing in people with a range of conditions e.g. chronic obstructive pulmonary disease, chronic fatigue, congestive heart failure and post-COVID syndrome ^15– 18^. There is evidence to support the rehabilitation of critically ill patients within ICU, but a paucity of literature to support rehabilitation following discharge from ICU and hospital ^19^. A 2015 Cochrane review (6 studies 483 adult ICU participants) found that evidence for the effectiveness of post-hospital discharge rehabilitation interventions for survivors of critical illness was inconclusive ^20^. Nonetheless, findings from qualitative studies suggest that whilst individuals’ needs after critical illness were multifaceted ^21^, experiences of participating in rehabilitation programmes were markedly positive across domains of health, wellbeing, and perceived rate and quality of recovery ^22^. Participants had previously been admitted to ICU and they emphasised the need for multicomponent rehabilitation as well as a more individualised approach ^22, 23^.

Identifying ways to support people returning home after a stay in ICU has been ranked a key priority for research by survivors, their families, and researchers ^12^. Feasible and alternative approaches to provide rehabilitation to patients are needed to reach all those who could benefit and to optimise geographic access ^24^. Technology-enabled care has been shown to be effective, cost efficient and accessible for delivery of rehabilitation in other illnesses and settings ^15, 25– 33^ but rehabilitation delivered via a remote platform needs to be tested in people after critical illness before widespread implementation in the NHS. Our proposed intervention includes strategies to improve recovery, collectively delivered in an efficient, centralised format for ease of user-accessibility in a modern health service. The rehabilitation intervention will be delivered remotely and attempt to accommodate accessibility issues, and promote ethnic and socioeconomic diversity.

We hypothesise that for people following a hospital admission that included admission to an ICU for a critical illness, a six-week remote multicomponent rehabilitation intervention improves health-related quality of life, physical function, fatigue, mood, and other health-related outcomes after eight weeks, compared to standard care. Our research question is **‘**What is the clinical and cost effectiveness of a remote multicomponent rehabilitation intervention in survivors of critical illness following discharge home from ICU, compared to standard care?’

## Objectives

a) To investigate the effects of a six-week remote multicomponent rehabilitation intervention compared to standard care on health-related quality of life at eight weeks post-randomisation

b) To investigate the short and longer term effects of a six-week remote multicomponent rehabilitation intervention compared to standard care on physical function, illness perceptions, fatigue, anxiety, depression, and adverse events at eight weeks and six months post-randomisation. 

c) To determine explanatory factors influencing outcomes via assessment of acceptability of the intervention and standard care and an embedded process evaluation. 

e) To evaluate the cost-effectiveness of the multicomponent rehabilitation intervention compared to standard care over six months follow-up.

## Methods

### Patient and Public Involvement

Effective PPI is at the heart of key decisions made during this trial. We have ensured partnership with our PPI members from the design stage, and this will continue through to completion and dissemination.

Our patient partners agreed the research question was important. Their input relating to the lived experience of the participant journey following critical illness confirmed the need to test an individualised multicomponent approach to rehabilitation.

They confirmed practical information e.g. access to computers, agreed the study primary outcome measure and advised about best approaches for seeking consent from a participant. We meet regularly with our iRehab Patient Advisory Group (PAG) for their input, and they have representation on the iRehab Trial Management Group to ensure their views are included in decisions about trial delivery. Training and support for all PPI members in the PAG is offered to help with understanding of trial and trial procedures, and to optimise opportunity for meaningful input. Examples of active contribution by the PAG include informing best practice guidance on lay language to explain the trial to patients and relatives when identifying patients for the trial, preparing materials including recruitment materials to optimise equity of access, advising on how the approach to delivery of the intervention needs to be refined at individual level to be cognisant of specific circumstances.

Our PAG are supporting plans for the trial dissemination of findings with other ICUs, clinicians, researchers, and patient groups. Their input will help ensure findings are presented in a format that is accessible to a wide audience and will include a range of opportunities for presenting trial results to patients and family members e.g. at support group meetings or via community or charity events, and use of social media. Key items relating to PPI in the iRehab trial will be reported using the Guidance for Reporting Involvement of Patients and the Public (version 2) checklist ^34^.

### Study design and setting

This is a pragmatic, randomised controlled, open-label, assessor blind, multicentre, clinical and cost effectiveness trial with internal pilot and embedded process evaluation. The study interventions will be delivered remotely via online video or by telephone in the participants’ own homes. Patients will be recruited from at least 30 NHS hospitals across UK.

This protocol is reported in accordance with recognised recommendations (SPIRIT) for reporting a clinical trial protocol ^35^.

### Population

People are eligible for inclusion if they meet all the following criteria: Aged ≥ 18 years; received continuous invasive mechanical ventilation for 48 hours or longer; are within 12 weeks following discharge home from hospital at time of consent; understand spoken English or have a family member/friend/other present to translate trial materials; able to participate in the intervention and with trial procedures e.g. using equipment such as computer or telephone.

Exclusion criteria are: Declined consent or unable to provide consent; Previous randomisation into the present trial; Participating in another rehabilitation or self-management support trial; Contra-indication to exercise; Severe mental health problems that preclude participation in a group intervention; Discharged to a rehabilitation unit, or care home with/without nursing care; Prisoners.

### Recruitment procedures

The inclusion/exclusion criteria will enrol a broad population who may benefit from the intervention. We will implement strategies to encourage inclusion, e.g. using local trial champions that understand the specific cultures within each hospital site; simplify recruitment and consent procedures and provide a translator for those not fluent in English. We have considered information from the INCLUDE project which includes suggestions about how to improve inclusion of groups that are at risk of being underserved ^36^. Any hospital that offers active structured rehabilitation programme as part of their care pathway will not be approached.

### Participant identification and consent process

Potential participants will be identified via screening by clinical teams/site trial champion at hospital sites. Potential participants may be identified through patient electronic databases at each of the trial sites, referrals or whilst patients attend hospital follow-up clinics. If a person would like to participate, their initial eligibility will be checked by a suitably trained member of the hospital research team listed on the trial delegation log. The research team member will ensure the potential participant has read the patient information leaflet (PIL), understands what is involved with the trial, is willing to be randomised and has had the chance to answer and discuss any questions before proceeding to consent.

After leaving hospital contact is usually by phone or an alternate method based on the patient’s preference (e.g. study information can be emailed, or posted). Consent to join the trial will be taken, by telephone or video call, once the participant has returned home, by an appropriately delegated member of the research team. This model for consent has become widely accepted in clinical trials ^37^. Participants will also be asked for their consent to be contacted at a later stage about an interview with a researcher.

People who self-refer will be considered for the trial provided eligibility criteria can be verified. The trial will be promoted though local/national media/social media, relevant charities and on the trial website. Eligibility for self-referred patients will be confirmed via hospital clinical or research teams with permission provided by the potential participants to source this confirmation, or by the potential participant directly contacting the hospital requesting they provide the confirmation.

### Trial interventions

**Figure 1.**
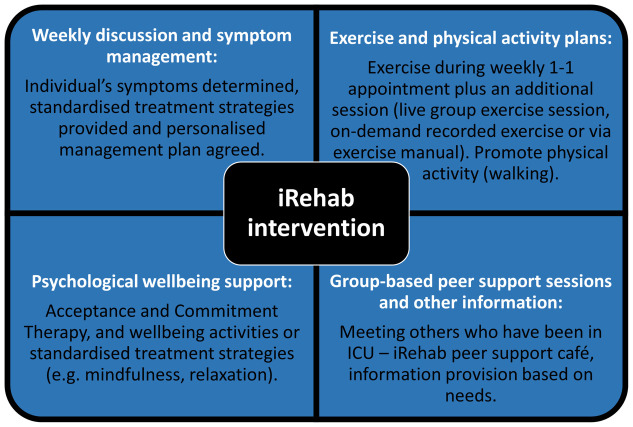
The four main components included in the iRehab intervention.

Rehabilitation intervention (iRehab Intervention): A patient-centred, structured, individually tailored, multi-component intervention delivered remotely to trial participants by a trained iRehab intervention team over six weeks ( https://openresearch.nihr.ac.uk/articles/5-64/v1). The rehabilitation package includes four core components ( Figure 1): 1. Weekly focused discussion and expert guidance to determine individual symptoms and management plans; 2. Exercise and physical activity 3. Psychological wellbeing support; 4: Group based peer support sessions and other information. These components are adapted and progressed according to individual ability and user accessibility, delivered via remote delivery by a core, trained intervention team. The intervention will be reported in accordance with the TIDieR checklist and guide ^38^. An overview is presented in Table 1.

**Table 1.** Overview of iRehab rehabilitation programme.

Timing	Session format	Content
Week 1	1-to-1 appointment, online	Explain programme Assess symptoms and identify needs Provide individual symptom management Agree and start exercise and activity plan Agree review appointments
	Independent or online group/recorded exercise session	Home exercise plan/attend group exercise session/access recorded exercise sessions
	Online peer support group (iRehab peer support cafe)	Attend iRehab peer support cafe
Weeks 2 to 6	1-to-1 appointment, online	Weekly review of symptoms and progression through treatment plans Continue needs assessment, symptom management and psychological support Complete live exercise and continue plan for weekly exercise/ physical activity Agree review appointments
	Independent or online group/recorded exercise session	Home exercise plan or attend group exercise session/access recorded exercise sessions
	Online peer support group (iRehab peer support cafe)	Attend iRehab peer support cafe
Week 6	Final 1-to-1 weekly session to include review and discharge	Encourage participant to continue with prescribed management plans Identify further sources of support Discharge from intervention.

**Format and mode of delivery:** Weekly one-to-one remote sessions with a trained iRehab specialist. Participants will be encouraged to attend a weekly group-based remote exercise session and a group-based remote peer support session (iRehab peer support Café).

The preferred mode of remote delivery will be agreed with the participant and potential barriers to implementation will be considered. Remote delivery will be facilitated by online platforms i.e. Microsoft Teams or Zoom, supported with video platform BEAM ^©^ or via telephone, and all participants will receive colour-printed study manual(s) by post which will be referred to during the remote sessions as needed. 

The iRehab intervention team will receive bespoke training and certification, and ongoing mentorship throughout the trial. To minimise performance bias in intervention delivery, the core components will be protocolised to guide overarching delivery, whilst still enabling flexibility in how components are applied to individual participants. Active monitoring and early feedback will be implemented to ensure intervention fidelity ^39– 41^

### Standard care

Standard NHS care, without active rehabilitation, will be the trial comparator. No further intervention will be offered after completion of baseline assessment and randomisation, other than usual NHS care. We will record healthcare resource use for all participants across the trial period.

### Data collection

Following consent, baseline demographic data collection will include medical history and co-morbidities, pre-ICU admission functional status, ICU admission illness severity using acute physiology and chronic health evaluation II (APACHE II) score and duration of ventilation, ICU and hospital stay. Clinical data will be recorded from hospital records. All outcome measures ( Table 2) will be collected at baseline, eight weeks, and six months post randomisation. Outcome measures will be collected remotely via electronic platform: participants will be sent an email and/or text link to access and complete these online, or we will post them and if required support completion by telephone, depending upon their preferred contact option. Healthcare and social services utilisation (e.g. healthcare appointments, accommodation status, carers, meals on wheels) will be collected at eight weeks and six months post randomisation.

**Table 2.** Schedule for data collection.

	Pre-randomisation		Post-randomisation			
	Screening	Baseline	Intervention	Follow-up		
Weeks ± No. Days	Weeks 0–16		Weeks 1–6	Week 8	8–26 weeks	Week 26
Check eligibility	X	X				
Consent participant		X				
Clinical data collection (by site)						
APACHE II score		X				
Medical history		X				
Co-morbidities		X				
Pre-ICU admission functional status		X				
Duration ventilation		X				
Length of ICU stay		X				
Length hospital stay		X				
Participant data collection						
EQ-5D-5L **		X		X		X
30sec Sit-to-Stand		X		X		X
BIPQ		X		X		X
FACT-F		X		X		X
HADS		X		X		X
Health and social care use				X		X
TFAQ				X		
**Randomisation**		R *				
Intervention delivery			X			
Process evaluation						
					X	
Participant self-report/Site data collection						
AEs		X	X	X		X
SAEs		X	X	X		X

*Randomisation after consent and participant baseline data collection. **The minimum core data set will include the EQ-5D-5L at eight weeks and six months Abbreviations BIPQ-Brief Illness Perception Questionnaire. FACIT-F - Functional Assessment of Chronic Illness Therapy – Fatigue. HADS - Hospital Anxiety and Depression Scale. TFAQ Theoretical Framework of Acceptability Questionnaire. AEs-Adverse Events. SAEs Serious Adverse Events

Participants will also be asked to complete the 30-second sit-to-stand test during an online video call (e.g. MS TEAMs) or telephone call with the independent assessor based at Warwick Clinical trials Unit (WCTU). Participants are screened prior to the test and provided with a pulse oximeter (by post) to measure oxygen saturations ^42^. If there are safety concerns then participants may be withdrawn from this element of outcome data collection) ^42, 43^.

### Outcome measures

***Primary outcome***

The trial primary outcome is HRQoL, measured using the EQ-5D-5L at eight weeks post-randomisation. The EQ-5D-5L is the recommended measure for assessing quality of life in core outcome sets for longer-term outcomes following respiratory failure and physical rehabilitation in critical illness ^44, 45^. Systematic reviews confirm the EQ-5D-5L to be similarly robust compared with other longer measures, such as the SF-36 ^46, 47^. The scale measures mobility, self-care, usual activities, pain/discomfort, and anxiety/depression from no problems to severe problems. These domains and the visual analogue scale (VAS 0–100mm) capturing health utility are widely used for health economic evaluation. The scale was responsive to change in a study assessing multicomponent rehabilitation in post-critical illness patients ^48^ and has been validated for telephone completion. Importantly, our PPI group endorsed quality of life as an important outcome to reflect recovery after critical illness.

***Secondary outcomes***

These include:

•   EQ-5D at six months ^44, 45^

•   Leg strength/exercise capacity (sit to stand test) ^42, 43^

•   Fatigue (FACIT-F) ^8^

•   Illness perception (Brief Illness Perception Questionnaire) ^49^

•   Emotional wellbeing (Hospital Anxiety and Depression Scale (HADS)) ^50^

•   Health and social care data

•   Intervention acceptability data will be collected using the theoretical framework of acceptability questionnaire (TFAQ) ^51^

•   Data on serious adverse events.

An overview of schedule of trial assessments is given in Table 2. Where relevant, appropriate permissions were obtained for use of outcome measures.

### Randomisation process

After consent and baseline data collection, participants will be randomised to either standard care only or the rehabilitation intervention. Randomisation will use a minimisation algorithm stratified by (i) hospital site and (ii) duration of continuous invasive mechanical ventilation (≤7 days : >7 days). The target allocation ratio will be 1.17: 1 (Intervention: Control) using a weighted random element to minimise the imbalance. The randomisation schedule will be generated using a computerised system developed by WCTU. Allocations will be done centrally by WCTU to ensure allocation concealment.

To maintain confidentiality, all Case Report Forms (CRFs), trial reports and communication regarding the trial will identify participants using unique identification numbers only. Treatment allocation will be concealed from the independent assessor performing outcome assessments and at follow-up participants will be asked to not reveal their allocation to the independent assessor. If allocation is revealed, the assessor will record this on the appropriate CRF and a second independent assessor will collect outcomes.

After randomisation, the participant’s General Practitioner (GP), and the consultant responsible for their in-patient care will be informed that they are taking part in iRehab trial. GPs will also be informed on the first occasion that a participant’s score for either anxiety or depression is ≥8 on the HADS questionnaire; these letters will include interpretation of HADS score so that the participants GP can provide follow up if appropriate. 

### Adherence

Adherence with trial interventions will be monitored throughout the trial. This is a complex multicomponent individualised intervention, and we will include a range of metrics to explore adherence. Uptake and adherence to the iRehab intervention will be evidenced by the number of sessions referred to and attended, over the six-week intervention delivery period ^52^. The categories for adherence will be finalised during the Process Evaluation (see next section). At a high level the expectation is that each participant will engage in at least one intervention session per week over the six-week period [where full adherence is defined as the participant has engaged with five or more rehabilitation sessions (out of six); partial adherence is defined as engaged with one to four sessions; and non-attendance defined as none]. Data will also be recorded on the inclusion of exercise components, use of strategies for symptom management, and engagement with psychology and peer support components.

### Process evaluation

The embedded process evaluation runs throughout the pilot and main trial ^40, 41, 53, 54^. This process evaluation is led by two trial investigators (JMB, BC) with experience of process evaluation and include the following:

(i) Trial monitoring data. The internal pilot includes assessing the opening of sites, and mapping the recruitment pathway using the Screened, Eligible, Approached, Randomised (SEAR) framework ^55^. We are monitoring the number of people approached, reasons for non-eligibility, rate of discontinuation from interventions and trial attrition to allow us to consider accessibility, reach and engagement ^55^

*(ii)* Intervention fidelity. Fidelity checklists developed in the pilot phase, aim to assess the fidelity of the different components of the intervention ^39– 41, 55^. We will assess certification of the intervention team to deliver the intervention, drift throughout the trial and delivery of retraining as required. Fidelity will be further explored in interviews.

(iii) User acceptability. The Theoretical Framework of Acceptability Questionnaire (TFAQ) will be used to assess the extent to which people delivering or receiving a healthcare intervention consider it to be acceptable, based on anticipated or experiential cognitive and emotional responses to the interventions ^51^. The TFAQ will be administered via an online link, post, email, by phone with a member of the research team or at the beginning of interviews.

(iv) Standard care. All sites will be asked for information on whether any structured rehabilitation is provided at the start and during the trial period. This will help us monitor usual care and whether this changed over time.

(v) Qualitative interviews. We will use a topic guide to explore participants’ experiences and opinions about acceptability of the intervention and standard care arms according to the TFAQ domains, and their experiences joining the trial and filling out questionnaires, and their recovery trajectory since discharge from hospital. Intervention arm participants will be asked what they liked and disliked about the intervention and feedback on particular programme components. Standard care participants will be asked about any follow-up care they received, and thoughts on the intervention. 

We will also use a topic guide to explore iRehab specialists’ experiences and opinions on training as an iRehab specialist, the iRehab intervention, intervention delivery and acceptability, thoughts on how to improve trial processes, and thoughts regarding the outcome of the trial. Where possible, we will interview study champions to explore their views about the remote intervention in practice and the trial process. Interviews will be audio recorded, transcribed and analysed. While each component will be undertaken and analysed separately, the findings will be triangulated to integrate the qualitative findings and the trial outcomes, and to help inform the interpretation of results ^53, 54^.

### Safety

Participant safety and well-being will be protected by implementing Warwick Clinical Trials Unit (WCTU) standard operating procedures for adverse event reporting.

Adverse Events (AEs) and Serious Adverse Events SAEs will be assessed and reported in keeping with regulatory requirements. An AE is defined as any untoward medical occurrence in a clinical trial participant which does not necessarily have a causal relationship with the treatment/intervention. Common AEs associated with exercise will not be recorded as AEs (Breathlessness, Light headedness/dizziness, Muscle stiffness/soreness, Tiredness/fatigue, Oxygen desaturation that resolves with appropriate management e.g. rest, breathing exercises, inhaled medications) and we have a defined escalation plan for participants who experience a fall or are at risk of mental health crisis. Any AEs that are not listed above will be assessed for seriousness and causality and will be reported accordingly.

SAEs that are common in this population and do not require reporting to the Clinical Trials Unit as an SAE for this trial are ‘Treatment which was elective or pre-planned, for a pre-existing condition’; these will be recorded as part of follow-up data collection in the participant questionnaires or in the relevant section(s) of the CRF (such as the hospital readmission CRF). Any event which fulfils the serious criteria will be reportable if it occurs, usually, within 48 hours of physical trial activity (physical trial activity can include, but is not limited to, exercise-based intervention sessions and sit-to-stand tests.)

### Trial oversight

Trial coordination will be based at Warwick Clinical Trials Unit (WCTU), University of Warwick. The multi-disciplinary Trial Management Group will oversee all aspects of trial design, delivery, quality assurance and data analysis. Significant issues arising from management meetings will be referred to the Trial Steering Committee or Investigators, as appropriate.

The Trial Steering Committee (TSC), with independent Chairperson, will monitor and supervise trial progress and advise on major trial decisions such as a need to change the protocol for any reason.

An independent Data Monitoring Committee (DMC) will review confidential trial reports containing recruitment, protocol compliance, safety data and interim assessments of outcome data. They will advise whether the trial should be amended or terminated based on any safety or ethical concerns. Ulster University will sponsor the study and along with WCTU will work with research sites to ensure local research governance.

### Data management and confidentially

Electronic Trial CRFs and participant questionnaires will be developed to collect all trial data Study documents and electronic identifiable information will have restricted access and be held on a secure, password-protected database. Names or addresses of participants will not be disclosed to anyone other than the staff involved in running the trial. A unique trial-specific identifier/code will be used on all documents other than the consent form and postal documents. The trial will be conducted in accordance with the current approved protocol, Good Clinical Practice (GCP), relevant data protection regulations and WCTU standard operating procedures (SOPs). Routine monitoring and risk assessment procedures will be conducted to protect patient safety and trial integrity.

A range of methods, including phone, text and email, will be used to capture trial data. Data will be collected for participants who discontinue or deviate from the protocol, unless they withdraw their consent. Notice of any deaths will be requested from the relevant hospital sites if this occurs.

### Sample size

The total sample size for iRehab will be **428 participants**. Using our primary outcome of EQ-5D-5L utility score at eight weeks, our target difference is 0.08 with a standard deviation of 0.2 (i.e. an effect size=0.4) ^56^. Assuming seven intervention specialists for the intervention delivery group and an inter-cluster correlation coefficient of 0.01 with 30% loss to follow up (LTFU), a total of 428 (control: 197 and intervention: 231) participants will be required ^57, 58^. The Morbeek’s formulation ^59^ was applied to allow for clustering in the intervention arm and thus an unequal randomisation ratio of 1: 1.17. A difference of 0.08 on the primary outcome is a justifiable clinical effect ^48, 56, 60, 61^.

### Internal pilot

The main trial includes a nine month internal pilot study with target recruitment of 101 patients over nine months from the first randomisation ^62, 63^. The pilot study follows the same processes described in the main trial. The internal pilot phase aims to test recruitment procedures, confirm and refine recruitment rates, assess protocol and intervention compliance, refine procedures for outcome data collection, and test procedures for referral to, delivery of and fidelity with the iRehab intervention ^53, 54, 61, 62, 64^. The recommended traffic light system will be used to guide decisions regarding progression of the trial based on pilot recruitment data [Please see Extended data file 4 for further details]. The main study proceeds following approval from independent committees and funder.

## Statistical analysis of efficacy and harms

### Statistics and data analysis

The main statistical analysis will be based on intention-to-treat. Data will be summarised and reported in accordance with CONSORT guidelines for RCTs ^63^.

## Statistical analysis plan

### Summary of baseline data and flow of patients

Baseline data will be summarised by treatment arm, using means, standard deviations (SD), medians, interquartile ranges (IQR) for continuous variables and frequencies and proportions for categorical variables. Screening data will be summarised and a CONSORT diagram will present participant flow throughout the trial ^63^.

### Primary and secondary outcome analysis

The primary outcome will be summarised using means, standard deviations, (SD) medians and interquartile ranges (IQR). Linear regression (heteroscedastic) model will be used to estimate the treatment effect with 95% confidence interval (CI), with and without adjustment for stratification variables, important patient-level covariates and practitioner/cluster effect, by intention-to-treat. If there is negligible clustering effect, then the usual linear regression will be used for the analysis. The impact of compliance will be assessed using CACE (complier average causal effect) analysis or other appropriate approach. Any continuous secondary outcomes will be assessed using linear regression (heteroscedastic) models and binary outcomes will be assessed using logistic regression models. Further details on the analysis of outcome measures and sensitivity analyses are given in the statistical analysis plan (provided separate to this paper).

### Subgroup analyses

Subgroup analysis specified *a priori* include (a) duration of mechanical ventilation (≤7 days vs >7 days) and (b) age (<= median age vs > median age) ^2, 6, 65^. The primary outcome will be examined in relation to these subgroups using an interaction in the model with treatment and sub-group effect.

### Health economic evaluation

A prospective within-trial economic evaluation, adhering to NICE Reference Case recommendations, from a NHS and personal social services perspective ^66^, will compare intervention with standard care. Healthcare resource use data will include health and social service use during the six-month follow-up period, collected via trial CRFs and costed using the most recently available published reference costs. Generic HRQoL will be assessed at baseline, eight weeks and six months using the EQ-5D-5L, with responses converted to health status scores using the UK value set recommended by NICE guidance at the time of analysis and sensitivity analyses conducted using alternative tariffs if this is likely to be useful for decision-making ^66^. Participant-level QALY estimates will be calculated using the trapezoidal rule. Analyses will explore and manage data missingness in line with the approach to missing or spurious data described within the statistical analysis or Health Economics Analysis Plan (HEAP) [a detailed description of health economic analyses is presented in a HEAP]. Every effort will be made to minimize missingness, but if appropriate, a suitable method such as multiple imputation will be used to account for missingness. Bootstrapped bivariate regression will estimate and visualize incremental cost-effectiveness ratios, acceptability curves and net monetary benefit. If findings are non-convergent at six months, we will explore the sensitivity of cost-effectiveness to extrapolation of costs and benefits beyond the trial time horizon, via a suitable decision model or parametric survival analysis model or extrapolation of net monetary benefit. Value of information analysis will be conducted to explore the sensitivity of health economic recommendations to additional research. Sensitivity analyses will also explore the impact of broadening the decision perspective beyond the NICE reference case to include indirect costs such as the impact on productivity. Additional secondary cost-effectiveness analyses will also explore the unit cost of any achieved reductions in fatigue, illness perceptions or anxiety/depression resulting from the intervention.

The study will seek to support a Study Within A Trial (SWAT) looking at the availability, accessibility and concordance with self-report of locally available electronic records on health care resource use, such as hospitalisations. If sufficient, such routine data are available, we will conduct an analysis of the sensitivity of health economic results to the source of resource use data.

## Discussion

Since this protocol was initiated, research continues to report the consequences of Post Intensive Care Syndrome (PICS) ^67, 68^. This trial provides an opportunity to deliver a definitive trial for the pragmatic evaluation of a remote multicomponent rehabilitation programme targeting survivors of critical illness following discharge from the ICU in whom post-hospital morbidity is substantial. This trial aims to investigate, in survivors of critical illness following discharge from hospital after an Intensive Care Unit (ICU) admission, the effects of a six-week remote multicomponent rehabilitation intervention compared to standard care on health-related quality of life at eight weeks post-randomisation.

## Ethics approval and consent to participate

**Ethics Reference Number**: London - Central Research Ethics Committee, 22/LO/0314.

Approval date: 18th May 2022

The trial will be conducted in conformance with the principles of the Declaration of Helsinki and to Good Clinical Practice (GCP) guidelines. It will also comply with all applicable UK legislation and Warwick Standard Operating Procedures (SOPs). All data will be stored securely and held in accordance with the UK GDPR and Common Law Duty of Confidentiality.

Consent to join the trial will usually be taken, by telephone or video call, once the participant has returned home. Consent will be taken by an appropriately delegated member of the research team. Potential participants will be asked to confirm they have read each of the consent items before agreeing to take part in the trial. A copy of the completed consent form will then be sent to the potential participant via email or post.

The trial protocol and related documents were approved by the London - Central Research Ethics Committee, 22/LO/0314 Approval date: 18th May 2022 Research Ethics Committee,

Approvals will be sought from each NHS Trust Research and Development office and sites will only be permitted to enrol patients into the trial once all required agreements are in place. Substantial protocol amendments (e.g., changes to eligibility criteria, outcomes, analyses) will be communicated by the trial team to relevant parties i.e., investigators, RECs, participants, NHS Trusts and trial registries. Annual reports will be submitted to the REC within 30 days of the anniversary date on which the favourable opinion was given, and annually until the trial is declared ended. The REC and sponsor will be notified of the end of the trial (whether the trial ends at the planned time or prematurely). The CI will submit a final report to the required authorities with the results, including any publications, within one year ending the trial.

**Sponsor:** Ulster University [email: e.bell2@ulster.ac.uk]. This role includes confirming that arrangements are in place for the research to begin, ensuring that the research protocol and processes are appropriate; confirming that ethical approval and other authorisations have been obtained before a study begins and ensuring that good practice arrangements are maintained for the duration of the study in relation to the conduct of the study, monitoring and reporting (including the immediate reporting of suspected unexpected serious adverse events or reactions)

## Trial registration

The trial is registered (04.07.2022) with the International Standard Randomised Controlled Trial Number (ISRCTN) Register ISRCTN11266403 https://doi.org/10.1186/ISRCTN11266403

**Protocol**: iRehab_Protocol_Version8.0_17.09.2024

## Trial status

Recruitment to this trial started on Dec 13 ^th^ 2022 and at the time of preparing this manuscript (05.02.2025), 381 patients had been recruited. We aim to complete recruitment by April 30 ^th ^2025 and analysis will commence after the 8 week follow up period is complete, and the database has been cleaned and locked. Further analysis will be after the 26 week follow up is completed. The current protocol version is Version8.0_17.09.2024.

## Data Availability

No data available at this protocol stage Following study completion, deidentified data sets generated will be available on request from WCTU Data Sharing Committee (DSC) ( WCTUDataAccess@warwick.ac.uk) and via a data-sharing agreement. All requests for data should be sent to wctu@datahs. Ulster University’s Research Portal : Remote multicomponent rehabilitation compared to standard care for survivors of critical illness after hospital discharge (iRehab): a protocol for a randomised controlled assessor-blind clinical and cost-effectiveness trial. Doi: https://doi.org/10.21251/044bc476-ce37-4604-9335-0b8ad8de28ca ^69^ 1.    iRehab CONSENT FORM: Remote multicomponent rehabilitation in survivors of critical illness after hospital discharge: The iRehab Trial 2.    iRehab Participant Information Leaflet: Remote multicomponent rehabilitation in survivors of critical illness after hospital discharge – the iRehab Trial 3.    iRehab internal pilot details Data are available under the terms of the Creative Commons Attribution 4.0 International license. Ulster University: SPIRIT checklist: Remote multicomponent rehabilitation compared to standard care for survivors of critical illness after hospital discharge (iRehab): a protocol for a randomised controlled assessor-blind clinical and cost-effectiveness trial. https://doi.org/10.21251/044bc476-ce37-4604-9335-0b8ad8de28ca

## References

[1] ICNARC Case Mix Programme Summary (2019–2020). Intensive Care National Audit and Research Centre, London, UK,2020. Reference Source 10.1186/cc6141 PMC2607109 19105799

[2] Dinglas VD Friedman LA Colantuoni E : Muscle weakness and 5–year survival in Acute Respiratory Distress Syndrome Survivors. *Crit Care Med.* 2017;45(3):446–453. 10.1097/CCM.0000000000002208 28067712 PMC5315580

[3] Hopkins RO Suchyta MR Kamdar BB : Instrumental activities of daily living after critical illness: a systematic review. *Ann Am Thorac Soc.* 2017;14(8):1332–1343. 10.1513/AnnalsATS.201701-059SR 28463657 PMC5566273

[4] Pandharipande PP Girard TD Jackson JC : Long-term cognitive impairment after critical illness. *N Engl J Med.* 2013;369(14):1306–16. 10.1056/NEJMoa1301372 24088092 PMC3922401

[5] Brodsky M Huang M Shanholtz C : Recovery from dysphagia symptoms after oral endotracheal intubation in acute respiratory distress syndrome survivors. A 5-year longitudinal study. *Ann Am Thorac Soc.* 2017;14(3):376–383. 10.1513/AnnalsATS.201606-455OC 27983872 PMC5427721

[6] Pfoh ER Wozniak AW Colantuoni E : Physical declines occurring after hospital discharge in ARDS survivors: a 5-year longitudinal study. *Intensive Care Med.* 2016;42(10):1557–1566. 10.1007/s00134-016-4530-1 27637716

[7] Oeyen SG Vandijck DM Benoit DD : Quality of Life after intensive care: a systematic review of the literature. *Crit Care Med.* 2010;38(12):2386–400. 10.1097/CCM.0b013e3181f3dec5 20838335

[8] Neufeld KJ Leoutsakos JMS Yan H : Fatigue symptoms during the first year Following ARDS. *Chest.* 2020;158(3):999–1007. 10.1016/j.chest.2020.03.059 32304774 PMC7478232

[9] Lone NI Lee R Salisbury L : Predicting risk of unplanned hospital readmission in survivors of critical illness: a population-level cohort study. *Thorax.* 2019;74(11):1046–1054. 10.1136/thoraxjnl-2017-210822 29622692

[10] Kamdar BB Huang M Dinglas VD : Joblessness and lost earnings after acute respiratory distress syndrome in a 1–year national multicenter study. *Am J Respir Crit Care Med.* 2017;196(8):1012–1020. 10.1164/rccm.201611-2327OC 28448162 PMC5649982

[11] Griffiths J Hatch RA Bishop J : An exploration of social and economic outcome and associated health-related quality of life after critical illness in general intensive care unit survivors: a 12–month follow-up study. *Crit Care.* 2013;17(3): R100. 10.1186/cc12745 23714692 PMC3706775

[12] Reay H Arulkumaran N Brett SJ : Priorities for future intensive care research in the UK: results of a James Lind Alliance Priority Setting Partnership. *J Intensive Care Soc.* 2014;15(4):288–296. 10.1177/175114371401500405 PMC5606390 28979474

[13] NICE: Rehabilitation after critical illness. NICE clinical guideline 83.National Institute for Health and Care Excellence, London, UK,2009. Reference Source 39480975

[14] Connolly B Milton-Cole R Adams C : Recovery, rehabilitation and follow-up services following critical illness: an updated UK national cross-sectional survey and progress report. *BMJ Open.* 2021;11(10): e052214. 10.1136/bmjopen-2021-052214 34607869 PMC8491421

[15] Puhan MA Gimeno-Santos E Cates CJ : Pulmonary Rehabilitation following exacerbations of chronic obstructive pulmonary disease. *Cochrane Database Syst Rev.* 2016;12(12): CD005305. 10.1002/14651858.CD005305.pub4 27930803 PMC6463852

[16] Larun L Brurberg KG Odgaard-Jensen J : Exercise therapy for chronic fatigue syndrome. *Cochrane Database Syst Rev.* 2019;10(10): CD003200. 10.1002/14651858.CD003200.pub8 31577366 PMC6953363

[17] Long L Mordi IR Bridges C : Exercise-based cardiac rehabilitation for adults with heart failure. *Cochrane Database Syst Rev.* 2019;1(1): CD003331. 10.1002/14651858.CD003331.pub5 30695817 PMC6492482

[18] McGregor G Sandhu H Bruce J : Clinical effectiveness of an online supervised group physical and mental health rehabilitation programme for adults with post-covid-19 condition (REGAIN study): multicentre randomised controlled trial. *BMJ.* 2024;384: e076506. 10.1136/bmj-2023-076506 38325873 PMC11134408

[19] Connolly B O'Neill B Salisbury L : Physical rehabilitation interventions for adult patients during critical illness: an overview of Systematic Reviews. *Thorax.* 2016;71(10):881–890. 10.1136/thoraxjnl-2015-208273 27220357 PMC5036250

[20] Connolly B Salisbury L O'Neill B : Exercise rehabilitation following intensive care unit discharge for recovery from critical illness. *Cochrane Database Syst Rev.* 2015;2015(6): CD008632. 10.1002/14651858.CD008632.pub2 26098746 PMC6517154

[21] King J O’Neill B Ramsay P : Identifying patients’ support needs following critical illness: a scoping review of the qualitative literature. *Crit Care.* 2019;23(1): 187. 10.1186/s13054-019-2441-6 31126335 PMC6533750

[22] Ferguson K Bradley JM McAuley DF : Patients’ perceptions of an exercise program delivered following discharge from hospital after critical illness (the revive trial). *J Intensive Care Med.* 2019;34(11–12):978–84. 10.1177/0885066617724738 28826281

[23] O'Neill B Green N Blackwood B : Recovery following discharge from intensive care: what do patients think is helpful and what services are missing? *PLoS One.* 2024;19(3): e0297012. 10.1371/journal.pone.0297012 38498470 PMC10947670

[24] Connolly B Denehy L : Hindsight and moving the needle forwards on rehabilitation trial design. *Thorax.* 2018;73(3):203–205. 10.1136/thoraxjnl-2017-210588 29138261

[25] NHS England Technology Enabled Care Services (TECS). [Last accessed 28.02.2025]. Reference Source

[26] Vasilopoulou M Papaioannou AI Kaltsakas G : Home-based maintenance tele-rehabilitation reduces the risk for acute exacerbations of COPD, hospitalisations and emergency department visits. *Eur Respir J.* 2017;49(5): 1602129. 10.1183/13993003.02129-2016 28546268

[27] Nakayama A Takayama N Kobayashi M : Remote cardiac rehabilitation is a good alternative of outpatient cardiac rehabilitation in the COVID-19 era. *Environ Health Prev Med.* 2020;25(1): 48. 10.1186/s12199-020-00885-2 32891113 PMC7474480

[28] Tew GA Bedford R Carr E : Community-based prehabilitation before elective major surgery: the PREP-WELL quality improvement project. *BMJ Open Qual.* 2020;9(1): e000898. 10.1136/bmjoq-2019-000898 32213551 PMC7206908

[29] Light K Bishop M Wright T : Telephone calls make a difference in home balance training outcomes: a randomized trial. *J Geriatr Phys Ther.* 2016;39(3):97–101. 10.1519/JPT.0000000000000069 26682647

[30] Dekker-van Weering M Jansen-Kosterink S Frazer S : User experience, actual use, and effectiveness of an information communication technology-supported home exercise program for pre-frail older adults. *Front Med (Lausanne).* 2017;4: 208. 10.3389/fmed.2017.00208 29250523 PMC5715376

[31] Holland AE Mahal A Hill CJ : Home-based rehabilitation for COPD using minimal resources: a randomised, controlled equivalence trial. *Thorax.* 2017;72(1):57–65. 10.1136/thoraxjnl-2016-208514 27672116 PMC5329049

[32] NSW Government Health: Delivering Pulmonary Rehabilitation via telehealth during COVID-19. Reference Source

[33] Connolly SB Kotseva K Jennings C : Outcomes of an integrated community-based nurse-led Cardiovascular Disease prevention programme. *Heart.* 2017;103(11):840–847. 10.1136/heartjnl-2016-310477 28255098 PMC5566096

[34] Staniszewska S Brett J Simera I : GRIPP2 reporting checklists: tools to improve reporting of patient and public involvement in research. *BMJ.* 2017;358: j3453. 10.1136/bmj.j3453 28768629 PMC5539518

[35] Chan AW Tetzlaff JM Altman DG : SPIRIT 2013 statement: defining standard protocol items for clinical trials. *Ann Intern Med.* 2013;158(3):200–7. 10.7326/0003-4819-158-3-201302050-00583 23295957 PMC5114123

[36] NIHR Improving inclusion of under-served groups in clinical research: guidelines form INCLUDE project.[Last accessed 11.03.2024]. Reference Source

[37] Gorman E Shankar-Hari M Hopkins P : Repair of Acute Respiratory Distress Syndrome by stromal cell administration in COVID-19 (REALIST-COVID-19): a structured summary of a study protocol for a randomised, controlled trial. *Trials.* 2020;21(1): 462. 10.1186/s13063-020-04416-w 32493473 PMC7267756

[38] Hoffmann TC Glasziou PP Boutron I : Better reporting of interventions: Template for Intervention Description and Replication (TIDieR) checklist and guide. *BMJ.* 2014;348: g1687. 10.1136/bmj.g1687 24609605

[39] Borrelli B : The assessment, monitoring, and enhancement of treatment fidelity in public health clinical trails. *J Public Health Dent.* 2011;71(s1):S52–S63. 10.1111/j.1752-7325.2011.00233.x 21499543 PMC3074245

[40] O’Shea O McCormick R Bradley J : Fidelity review: a scoping review of the methods used to evaluate treatment fidelity in behavioural change interventions. *Phys Ther Rev.* 2016;21(3–6):207–214. 10.1080/10833196.2016.1261237

[41] Bradley JM Hutchings M Arden MA : A RCT to explore the effectiveness of supporting adherence to nebuliser medication in adults with Cystic Fibrosis: fidelity assessment of study interventions. *BMC Pulm Med.* 2024;24(1): 148. 10.1186/s12890-024-02923-z 38509494 PMC10956306

[42] Bowman A Denehy L Benjemaa A : Feasibility and safety of the 30–second Sit-to-Stand test delivered via telehealth: an observational study. *PM R.* 2023;15(1):31–40. 10.1002/pmrj.12783 35138036

[43] Morita AA Bisca GW Machado FVC : Best protocol for the Sit-to-Stand test in subjects with COPD. *Respir Care.* 2018;63(8):1040–1049. 10.4187/respcare.05100 29789413

[44] Needham DM Sepulveda KA Dinglas VD : Core outcome measures for clinical research in Acute Respiratory Failure survivors. An international modified Delphi consensus study. *Am J Respir Crit Care Med.* 2017;196(9):1122–1130. 10.1164/rccm.201702-0372OC 28537429 PMC5694837

[45] Connolly B Denehy L Hart N : Physical Rehabilitation Core Outcomes In Critical illness (PRACTICE): protocol for development of a Core Outcome Set. *Trials.* 2018;19(1): 294. 10.1186/s13063-018-2678-4 29801508 PMC5970518

[46] Robinson KA Davis WE Dinglas VD : A systematic review finds limited data on measurement properties of instruments measuring outcomes in adult Intensive Care Unit survivors. *J Clin Epidemiol.* 2017;82:37–46. 10.1016/j.jclinepi.2016.08.014 27865899 PMC5325798

[47] Gerth AMJ Hatch RA Young JD : Changes in health-related quality of life after discharge from an Intensive Care Unit: a systematic review. *Anaesthesia.* 2019;74(1):100–108. 10.1111/anae.14444 30291744 PMC6586053

[48] McPeake J Shaw M Iwashyna TJ : Intensive care Syndrome: Promoting Independence and Return to Employment (InS:PIRE). Early evaluation of a complex intervention. *PLoS One.* 2017;12(11): e0188028. 10.1371/journal.pone.0188028 29186177 PMC5706708

[49] Broadbent E Petrie KJ Main J : The Brief Illness Perception Questionnaire. *J Psychosom Res.* 2006;60(6):631–37. 10.1016/j.jpsychores.2005.10.020 16731240

[50] Zigmond AS Snaith RP : The hospital anxiety and depression scale. *Acta Psychiatr Scand.* 1983;67(6):361–370. 10.1111/j.1600-0447.1983.tb09716.x 6880820

[51] Sekhon M Cartwright M Francis JJ : Development of a theory-informed questionnaire to assess the acceptability of healthcare interventions. *BMC Health Serv Res.* 2022;22(1): 279. 10.1186/s12913-022-07577-3 35232455 PMC8887649

[52] McDowell K O'Neill B Blackwood B : Effectiveness of an exercise programme on physical function in patients discharged from hospital following critical illness: a randomised controlled trial(the REVIVE trial). *Thorax.* 2017;72(7):594–95. 10.1136/thoraxjnl-2016-208723 27852953

[53] Skivington K Matthews L Simpson SA : A new framework for developing and evaluating complex interventions: update of Medical Research Council guidance. *BMJ.* 2021;374: n2061. 10.1136/bmj.n2061 34593508 PMC8482308

[54] Moore GF Audrey S Barker M : Process evaluation of complex interventions: Medical Research Council guidance. *BMJ.* 2015;350: h1258. 10.1136/bmj.h1258 25791983 PMC4366184

[55] O'Neill B O'Shea O McDonough S : Clinician-facilitated physical activity intervention versus Pulmonary Rehabilitation for improving physical activity in COPD: a feasibility study. *COPD.* 2018;15(3):254–264. 10.1080/15412555.2018.1486396 30183414

[56] Batterham AM Bonner S Wright J : Effect of supervised aerobic exercise rehabilitation on physical fitness and quality-of-life in survivors of critical illness: an exploratory minimized controlled trial (PIX study). *Br J Anaesth.* 2014;113(1):130–37. 10.1093/bja/aeu051 24607602 PMC4062299

[57] Yohannes AM Casaburi R Dryden S : Predictors of premature discontinuation and prevalence of dropouts from a Pulmonary Rehabilitation program in patients with Chronic Obstructive Pulmonary Disease. *Respir Med.* 2022;193: 106742. 10.1016/j.rmed.2022.106742 35091205

[58] Busch AM Scott-Sheldon LAJ Pierce J : Depressed mood predicts Pulmonary Rehabilitation completion among women, but not men. *Respir Med.* 2014;108(7):1007–1013. 10.1016/j.rmed.2014.04.010 24820243 PMC4116192

[59] Moerbeek M Wong WK : Sample size formulae for trials comparing group and individual treatments in a multilevel model. *Stat Med.* 2008;27(15):2850–64. 10.1002/sim.3115 17960589

[60] Pickard AS Neary MP Cella D : Estimation of minimally important differences in EQ-5D utility and VAS scores in cancer. *Health Qual Life Outcomes.* 2007;5: 70. 10.1186/1477-7525-5-70 18154669 PMC2248572

[61] Cocks K Torgerson DJ : Sample size calculations for pilot randomized trials: a confidence interval approach. *J Clin Epidemiol.* 2013;66(2):197–201. 10.1016/j.jclinepi.2012.09.002 23195919

[62] Avery KNL Williamson PR Gamble C : Informing efficient Randomised Controlled Trials: exploration of challenges in developing progression criteria for internal pilot studies. *BMJ Open.* 2017;7(2): e013537. 10.1136/bmjopen-2016-013537 28213598 PMC5318608

[63] Moher D Schulz KF Altman DG : The CONSORT statement: revised recommendations for improving the quality of reports of parallel-group randomised trials. *Lancet.* 2001;357(9263):1191–4. 11323066

[64] Wilson C Rooshenas L Paramasivan S : Development of a framework to improve the process of recruitment to Randomised Controlled Trials (RCTs): the SEAR (Screened, Eligible, Approached, Randomised) framework. *Trials.* 2018;19(1): 50. 10.1186/s13063-017-2413-6 29351790 PMC5775609

[65] Herridge MS Chu LM Matte A : The RECOVER program: disability risk groups and 1-year outcome after 7 or more days of Mechanical Ventilation. *Am J Respir Crit Care Med.* 2016;194(7):831–44. 10.1164/rccm.201512-2343OC 26974173

[66] NICE technology appraisal guidance.Last accessed 26.01.2022. Reference Source

[67] Hiser SL Fatima A Ali M : Post-Intensive Care Syndrome (PICS): recent updates. *J Intensive Care.* 2023;11(1): 23. 10.1186/s40560-023-00670-7 37221567 PMC10202754

[68] Cagino LM Seagly KS McSparron JI : Survivorship after critical illness and Post-Intensive Care Syndrome. *Clin Chest Med.* 2022;43(3):551–561. 10.1016/j.ccm.2022.05.009 36116822

[69] O'Neill B Bradley JM Connolly B : SPIRIT checklist: remote multicomponent rehabilitation compared to standard care for survivors of critical illness after hospital discharge (iRehab): a protocol for a randomised controlled assessor-blind clinical and cost-effectiveness trial. 2025. 10.21251/044bc476-ce37-4604-9335-0b8ad8de28ca PMC12120417 40443419

