# Supplementary material for: Remote multicomponent rehabilitation compared to standard care for survivors of critical illness after hospital discharge (iRehab): a protocol for a randomised controlled assessor-blind clinical and cost-effectiveness trial
Source: NIHR Open Res. 2025 Aug 22;5:29. Originally published 2025 Apr 10. [Version 2] doi: 10.3310/nihropenres.13910.2 (PMC12120417; doi:10.3310/nihropenres.13910.2)
Supplement: Peer review history

# Reviewer report: Reviewer response for version 1

**DOI:** 10.3310/nihropenres.15118.r35316

**Published:** 2025-05-29

**Authors:** Heather K O'Grady

**Affiliations:** 1 McMaster University, Hamilton, Ontario, Canada; 2 Niagara Health Knowledge Institute, Niagara Health System (Ringgold ID: 27363), Saint Catharines, Ontario, Canada

O’Neill and colleagues report a protocol for a multi-component rehabilitation intervention (iRehab) for survivors of critical illness. The authors provide a detailed description of the trial rationale, design and analysis plan.

 Thank you very much for the opportunity to review this manuscript. As ICU survivorship increases, there is an increasing need to identify interventions to support survivors experiencing important post-ICU morbidity. This work adds to a growing body of literature exploring rehabilitation strategies to mitigate PICS. This work is novel and addresses an important question in the field of ICU rehabilitation. Particularly, the authors are to be congratulated for a comprehensive trial design, with the inclusion of critical elements that will contribute to its interpretation including patient and public involvement and an embedded pilot, process evaluation, and economic evaluation. This protocol manuscript is very well written and transparently reported.

 Below, I will have included some minor comments for consideration.

**Methods**

1. Participant Identification and Consent - Can the authors please clarify how potential participants are contacted after they are screened by clinical teams/site trial champion at hospital sites? (i.e., phone, email)
2. Trial Interventions - Was there a pre-specified number of minimum or maximum participants for the “group-based” activities (i.e., exercise session and peer support)?
3. Trial Interventions - If pre-specified, it would be helpful to understand additional details of intervention components to allow replication (i.e., parameters of the exercise interventions, structure of the peer support sessions). If not pre-specified, it might be helpful to state this and include these details in the results manuscript (as suggested with the TIDieR checklist).
4. Secondary Outcomes - The 30s STS might be better characterized as a measure of lower extremity strength (vs. a global measure of physical strength) – it is also a measure of exercise capacity, both of which are supported by the included reference #42.
5. Trial Oversight - I think there is a sentence cut-off at the end of this paragraph. Reads "si".
6. Internal Pilot - Were there pre-specified criteria to help determine whether the main study would proceed following the internal pilot? If so, it would be helpful to report these for transparency.
7. Process Evaluation - The authors might want to consider including an implementation framework (e.g., the Consolidated Framework for Implementation Research; https://doi.org/10.1186/s13012-022-01245-0) to help guide the process evaluation analysis.

Is the study design appropriate for the research question?

Yes

Is the rationale for, and objectives of, the study clearly described?

Yes

Are sufficient details of the methods provided to allow replication by others?

Yes

Are the datasets clearly presented in a useable and accessible format?

Not applicable

Reviewer Expertise:

Knowledge Translation, Patient and Family Engagement, Research Methods, ICU-Based Rehabilitation

I confirm that I have read this submission and believe that I have an appropriate level of expertise to confirm that it is of an acceptable scientific standard.

# Reviewer report: Reviewer response for version 1

**DOI:** 10.3310/nihropenres.15118.r35191

**Published:** 2025-05-06

**Authors:** Kirby Mayer

**Affiliations:** 1 University of Kentucky, Kentucky, USA

Dear authors, thank you for your dedication to ICU Recovery and PICS. The manuscript is well-structured and well-written. The approach to post-ICU interventions is clinically meaningful. The multi-component intervention strategy aimed at improving HrQOL is very important. I am also very appreciative to read your focus and plan for intervention fidelity and process evaluation. My main comments are related to the approach to interventions as it may be very challenging to replicate the study with limited descriptions of the interventions.  

 1.) The **Trial Interventions** section state that interventions are "details are reported separately."  Besides Table 1 - are more details about the intervention available? Specifically, what is "targeted" and "individualized" exercise. The table states a home exercise plan, but how are exercises individualized i.e. physiologically based on heart rates; functional status; domain deficits i.e. aerobic vs muscle strength training?  If it is "group-based" as mentioned in format then how is exercise targeted/individualized? 

 2) Symptoms and management plans - is it possible to provide additional explanations? For example, if pain is a symptom is the team providing pain management strategies, analgesics, etc. As a clinician in an ICU Recovery Clinic, I understand the spectrum of symptoms and treatment for patients, and your approach should be celebrated. But the methods lack detail in the intervention delivery that would prevent reproducibility of interventions - I would recommend elaborating when possible. For example, if a medical provider is selecting any management or drug available, then could this be stated: "After symptom assessment, an iREHAB team member provided treatment based on their clinical judgement. Treatment approaches included, but not limited to XXX, XXX, XX>.. 

 3)  Psychological well-being support - is this delivered via a trained/licensed psychologist? is this general support? is this medications?  - can more detail be provided to describe the approach? 

 - The intervention overview is helpful, but I am not completely sure what is being provided. Perhaps, I missed a table or supplemental file.  I would suggest that details in the intervention will enhance the rigor and reproducibility in reporting. 

 Minor Comments:

 1.) Can you elaborate on the exclusion - contra-indication to exercise? will this be based on exercise guidelines or clinical practice guidelines? 

 2.) who are the iRehab intervention team members that are described in the format and mode of delivery - what disciplines? did they have ICU or post ICU experience? can details be provided about how they were selected? 

 3.) Remote delivery enhances implementation for majority of individuals; what about individuals who lack access to internet or telephone? will they be excluded? will you address for technological barriers in adherence plan?

 4) Can the selection of 6-week approach be justified? was this based on literature, pilot data, or simply feasibility of delivery? 

 5.) Since the study was registered in April 2022 and already enrolled 381 patients, were there any modifications to the protocol that should be acknowledged or transparent? It appears the current protocol is on Version 8.0. 

 6.) Subgroup analyses - is the team planning for any sub-analyses based on diagnosis or comorbidities. There have been several investigations that support that comorbidity alone can influence the primary outcome of HrQOL  (Orwelius et al Critical Care 2024) (Jones et al CCMEd 2023);

Is the study design appropriate for the research question?

Yes

Is the rationale for, and objectives of, the study clearly described?

Yes

Are sufficient details of the methods provided to allow replication by others?

Partly

Are the datasets clearly presented in a useable and accessible format?

Yes

Reviewer Expertise:

Post-Intensive Care Syndrome; Rehabilitation; Skeletal Muscle Dysfunction; Emotional and Cognitive Health; Social Determinants of Health

I confirm that I have read this submission and believe that I have an appropriate level of expertise to confirm that it is of an acceptable scientific standard.
